# Serum Level of Soluble CD163 May Be a Predictive Marker of the Effectiveness of Nivolumab in Patients With Advanced Cutaneous Melanoma

**DOI:** 10.3389/fonc.2018.00530

**Published:** 2018-11-19

**Authors:** Taku Fujimura, Yota Sato, Kayo Tanita, Yumi Kambayashi, Atsushi Otsuka, Yasuhiro Fujisawa, Koji Yoshino, Shigeto Matsushita, Takeru Funakoshi, Hiroo Hata, Yuki Yamamoto, Hiroshi Uchi, Yumi Nonomura, Ryota Tanaka, Megumi Aoki, Keisuke Imafuku, Hisako Okuhira, Naoko Wada, Hiroyuki Irie, Takanori Hidaka, Akira Hashimoto, Setsuya Aiba

**Affiliations:** ^1^Department of Dermatology, Tohoku University Graduate School of Medicine, Sendai, Japan; ^2^Department of Dermatology, Kyoto University Graduate School of Medicine, Kyoto, Japan; ^3^Department of Dermatology, Faculty of University of Tsukuba, Tsukuba, Japan; ^4^Department of Dermatology, Tokyo Metropolitan Cancer and Infectious Disease Center Komagome Hospital, Tokyo, Japan; ^5^Department of Dermato-Oncology, Dermatology, National Hospital Organization Kagoshima Medical Center, Kagoshima, Japan; ^6^Department of Dermatology, Keio University School of Medicine, Tokyo, Japan; ^7^Department of Dermatology, Hokkaido University Graduate School of Medicine, Sapporo, Japan; ^8^Department of Dermatology, Wakayama Medical University, Wakayama, Japan; ^9^Department of Dermatology, Kyushu University Graduate School of Medicine, Fukuoka, Japan

**Keywords:** sCD163, TAMs, melanoma, nivolumab, prediction of efficacy

## Abstract

Antibodies against programmed cell death protein 1, such as nivolumab and pembrolizumab, are widely used for treating various cancers, including advanced melanoma. Nivolumab significantly prolongs survival in patients with metastatic melanoma, and sequential administration with lipilimumab may improve outcomes when switched at the appropriate time. Biomarkers are therefore needed to evaluate nivolumab efficacy soon after first administration. This study analyzed serum levels of soluble cluster of differentiation 163 (sCD163) in 59 cases of advanced cutaneous melanoma and 16 cases of advanced mucosal melanoma treated using nivolumab. Serum levels of sCD163 were significantly increased after 6 weeks in responders compared to non-responders after initial administration of nivolumab for cutaneous melanoma. In contrast, no significant difference between responders and non-responders was seen among patients with non-cutaneous melanoma. These results suggest that sCD163 may be useful as a biomarker for selecting patients with advanced cutaneous melanoma most likely to benefit from anti-melanoma immunotherapy.

## Introduction

The programmed cell death protein 1/programmed death-ligand 1 (PD-1/PD-L1) pathway plays a critical role in tumor immune response. Anti-PD-1 antibodies such as nivolumab and pembrolizumab are thus in wide use for the treatment of various cancers, including advanced melanoma (1, 2). Nivolumab significantly prolongs survival in patients with metastatic melanoma (1), and co-administration with ipilimumab also leads to improved outcomes (1–3). Ipilimumab is a fully humanized immunoglobulin (Ig)G1 monoclonal antibody that blocks cytotoxic T-lymphocyte antigen (CTLA-4) to activate and increase T cells, and suppress the function of regulatory T cells (Tregs) (4). Previous reports have suggested ipilimumab is useful for treating advanced melanoma, particularly in combination with other anti-melanoma reagents (1–3, 5). Moreover, combination therapy using nivolumab and ipilimumab reportedly increases the response rate for untreated metastasis of melanoma to the brain (6). However, the efficacy of ipilimumab in patients with nivolumab-resistant melanoma is extremely low after objective tumor progression (7). Because both co-administration of nivolumab and ipilimumab and sequential administration of nivolumab and ipilimumab with a planned switch leads to a high frequency of immune-related adverse events (irAEs) in patients with advanced melanoma (1, 3), determining the efficacy of nivolumab monotherapy before the planned switch from nivolumab to ipilimumab is important.

Tumor-associated macrophages (TAMs) are characterized by their heterogeneity and plasticity, and may be functionally reprogrammed to polarized phenotypes by exposure to cancer-related factors, stromal factors, or infection, leading to the production of various chemokines that play significant roles in maintaining the tumor microenvironment (8–13). Because PD-1 expression in TAMs is one of the key factors in M2 macrophage polarization (14), administration of an anti-PD1 antibody might repolarize TAMs, leading to TAM activation in melanoma patients. Because the main population of TAMs in skin cancer is CD163^+^ M2 macrophages, and soluble cluster of differentiation 163 (sCD163) is a TAM marker that appears in the serum as a result of proteolytic shedding (15), we hypothesized that serum sCD163 may offer a predictive marker for the efficacy of nivolumab in the early stage of disease. This study analyzed serum levels of sCD163 in 59 cases of advanced cutaneous melanoma and 16 cases of advanced mucosal melanoma treated with nivolumab.

## Patients and methods

### Ethics statement for human experiments

The protocol for this human study was approved by the ethics committee of Tohoku University Graduate School of Medicine, Sendai, Japan (Permit No: 2017-1-064). All methods were performed in accordance with the relevant guidelines and regulations. All patients provided written informed consent.

### Patients

Data from patients treated with nivolumab were collected from eight clinical sites in Japan. Patients were eligible if they had unresectable stage III melanoma, the tumor was resectable but the patient had declined resection, or if the patient had stage IV melanoma with accessible cutaneous, subcutaneous, and/or nodal lesions (staging was performed according to the AJCC Staging Manual, 7th edition, 2011). All patients received 2 mg/kg of nivolumab followed by a 3-week rest period or 3 mg/kg of nivolumab followed by 2 weeks of rest, both of which are approved dosing schedules in Japan. Serum from patients was obtained on days 0 and 42.

### Serum levels of sCD163

On days 0 and 42 after nivolumab administration, we stored the serum and analyzed serum levels of soluble sCD163 by enzyme-linked immunoassay (ELISA) according to the protocol provided by the manufacturer (catalog number DY1607; R&D Systems, Minneapolis, MN). Data from each donor were obtained as the mean of duplicate assays. Serum levels on day 42 were compared to baseline (day 0) and statistically analyzed.

### Statistical methods

Receiver operating characteristic (ROC) curves were applied to calculate cut-off values for serum levels of sCD163 and areas under the curve (AUCs). Cut-offs were determined using Youden's index (16) (sensitivity + specificity −1) to determine the point of maximum index value. ROC curves were established to evaluate serum levels of sCD163 in patients administered nivolumab. For a single comparison between two groups, the Mann-Whitney U-test was used. Levels of significance were set at p < 0.01. All statistical analyses were performed using JMP version 14.1 software (SAS Institute, Tokyo, Japan).

## Results

### Patients

We collected data from 75 melanoma patients treated with nivolumab, including 59 patients with cutaneous melanoma (Table 1) and 16 patients with non-cutaneous (e.g., digestive tract, vagina, nasal cavity) melanoma (Table 2). Mean patient age was 68.0 years for cutaneous melanoma (range, 31–93 years) and 66.0 years for non-cutaneous melanoma (range, 54–82 years). The percentages of male patients with cutaneous and non-cutaneous melanoma were 57.6 and 56.3%, respectively. The most common primary tumor site was the extremities (55.9%), followed by the head and neck (15.3%), trunk (11.9%), mucosal origin (8.5%), and unknown origin (8.5%).

**Table 1:** Characteristics and serum levels of sCD163 in patients with cutaneous melanoma.

	**Age (y)**	**Sex**	**Location**	**Body weight (kg)**	**Prior systemic therapy**	**Efficacy at 3 months**	**Increase of sCD163**	**Change ratio of sCD163 (%)**
No. 1	61–70	M	Extremities	63.2	DAV+IFN-β	PR	11.556	149
No. 2	61–70	M	Extremities	48	–	PR	50.8525	227.8
No. 3	81–90	F	Extremities	48.7	DPCP	PR	12.676	152.7
No. 4	61–70	M	Extremities	70.4	DAV+IFN-β	PR	13.27122	146.2
No. 5	61–70	M	Extremities	58.5	DAV+IFN-β	PR	7.17709	127
No. 6	61–70	M	Lip	69.8	DAV+IFN-β	PR	5.834	123.1
No. 7	71–80	M	Trunk	59.4	–	PR	21.49956	133.4
No. 8	71–80	F	Extremities	68	–	PR	−12.9704	81.6
No. 9	31–40	F	Extremities	55.4	DTIC+IFN-β	PR	24.5042321	189.5
No. 10	71–80	M	Extremities	61.2	–	PR	3.36944	109.5
No. 11	51–60	F	Extremities	41.7	–	CR	2.109187	170.7
No. 12	81–90	M	Unknown	68.3	–	PR	3.07118	111.3
No. 13	71–80	F	Extremities	50.1	IFN-α	PR	35.7011169	158.5
No. 1	51–60	M	Trunk	68.7	–	SD	1.5625	107.5
No. 2	31–40	F	Extremities	60.8	CBDCA+PTX	PD	−9.6755	82.4
No. 3	61–70	M	Trunk	62	DTIC+IFN-β	PD	2.884	109.6
No. 4	81–90	F	Extremities	44.9	IFN-α	PD	−0.333	98.7
No. 5	71–80	M	Head and neck	59.2	IFN-β	SD	0.998	103.6
No. 6	81–90	F	Trunk	41.9	IFN-β	PD	6.76453	116.9
No. 7	91–100	M	Extremities	55	IFN-β	PD	4.02405	113
No. 8	71–80	M	Extremities	85.9	IFN-β	SD	−5.10307	89.9
No. 9	31–40	M	Extremities	73	TMZ	PD	−16.806	67.8
No. 10	61–70	M	Extremities	55	anti-CCR4 Abs	PD	2.706	115.4
No. 11	61–70	F	Extremities	66.3	DAV+IFNβ	PD	0.653	103.4
No. 12	61–70	M	Head and neck	55	DTIC	PD	−0.094	99.6
No. 13	61–70	M	Extremities	115.4	DAV+IFN-β	SD	0.177	100.5
No. 14	61–70	M	Extremities	77.6	IFN-β	PD	−61.59461	35.5
No. 15	81–90	F	Unknown	47.8	–	SD	−6.54815	86.5
No. 16	71–80	M	Extremities	54.4	IFN-β	SD	−4.74553	80.8
No. 17	61–70	F	Trunk	46.5	–	PD	17.3346	140.4
No. 18	71–80	M	Extremities	53.7	–	PD	−1.595225	93
No. 19	51–60	F	Head and neck	49.2	DTIC+IFN-β	PD	5.457395	115.9
No. 20	31–40	M	Trunk	78.8	IFN-α	SD	−12.615365	53.7
No. 21	61–70	F	Trunk	48.8	IFN-β	PD	−1.7178157	96.2
No. 22	31–40	F	Head and neck	52.3	IFN-β	PD	3.88229	120.1
No. 23	71–80	F	External genitalia	59.1	–	SD	−3.34502	92.1
No. 24	71–80	F	External genitalia	53.4	IFN-β	SD	−7.38773	70.5
No. 25	61–70	F	Extremities	47.0	–	PD	−4.11263	64
No. 26	61–70	M	Unknown	53.6	–	PD	−3.78005	75.6
No. 27	61–70	F	Extremities	60.9	IFN-β	PD	−0.54818	95.9
No. 28	61–70	F	Head and neck	41.6	IFN-α	PD	−1.456879	80.8
No. 29	71–80	M	Trunk	56	–	PD	−0.71335	77.2
No. 30	41–50	F	External genitalia	69.2	–	PD	−0.170996	94.4
No. 31	71–80	F	Extremities	53.3	–	PD	−0.682222	85.8
No. 32	61–70	M	Extremities	61	–	SD	2.202349	183.2
No. 33	71–80	M	Unknown	55	–	PD	0.328041	107.4
No. 34	61–70	F	Extremities	68	–	SD	0.228017	106.3
No. 35	71–80	M	Extremities	59.3	IFN-β	SD	−1.7178157	96.2
No. 36	61–70	M	Unknown	49.1	–	PD	0.428485	117.4
No. 37	61–70	M	Head and neck	61	–	SD	0.164125	110.7
No. 38	41–50	F	External genitalia	56	–	SD	1.268748	247.2
No. 39	71–80	F	Extremities	47	–	PD	−0.721186	86.1
No. 40	41–50	M	Head and neck	103	DTIC	PD	−0.111639	98
No. 41	61–70	M	Extremities	73	IFN-β	SD	0.421323	113.8
No. 42	31–40	M	Extremities	63.5	–	PD	0.9897167	102.1
No. 43	71–80	F	Extremities	66.2	–	SD	7.3643392	116.5
No. 44	71–80	M	Extremities	63.7	IFN-β	PD	−7.0989841	91.9
No. 45	71–80	M	Head and neck	57.4	–	PD	−0.5957191	98.4
No. 46	41–50	M	Extremities	62.7	IFN-β	PD	2.4247196	105.7

*CBDCA, carboplatin; PTX, paclitaxel; DTIC; dacarbazine; TMZ, temozolomide; DAV, dacarbazine + nimustine hydrochloride + vincristine. Serum level changes of sCD163 from each patient (n = 59) between day 42 and day 0 were examined by ELISA. Data for each donor represent the means of duplicate assays*.

**Table 2:** Characteristics and serum levels of sCD163 in patients with non-cutaneous melanoma.

	**Age (y)**	**Sex**	**Location**	**Body weight (kg)**	**Prior systemic therapy**	**Efficacy at 3 months**	**Increase of sCD163**	**Change ratio of sCD163 (%)**
No. 1	61–70	M	Digestive duct	62	–	PR	−0.444523	81.5
No. 2	61–70	F	Palate	52.8	–	PR	−0.799493	77.6
No. 3	61–70	M	Paranasal	71	–	PR	−1.803397	62.8
No. 4	61–70	F	Vagina	52.5	–	PR	0.469173	114.6
No. 1	61–70	M	Vagina	53.4	–	SD	−17.52087	78.7
No. 2	61–70	M	Vagina	80.4	–	PD	49.95895	134.6
No. 3	51–60	F	Conjunctiva	84	–	SD	8.27076	115.4
No. 4	81–90	M	Digestive duct	49.5	–	PD	19.6379	158.1
No. 5	61–70	F	Digestive duct	49.3	–	SD	20.26377	155.2
No. 6	71–80	F	Vagina	50.1	–	PD	28.525981	192.1
No. 7	61–70	M	Nasal cavity	47.2	DTIC	PD	17.0558	132.6
No. 8	61–70	F	Vagina	53.1	DAV + IFN-β	PD	−17.787	64.2
No. 9	61–70	M	Paranasal	56	DTIC	PD	0.16109	132.6
No. 10	51–60	M	Palate	52.9	–	PD	1.086	107.3
No. 11	71–80	M	Nasal cavity	53.1	DAV + IFN-β	SD	2.52838	107.6
No. 12	71–80	F	Nasal cavity	53	–	SD	−0.345647	89.4

*DAV, dacarbazine + nimustine hydrochloride + vincristine; DTIC, dacarbazine Serum level changes of sCD163 from each patient (n = 16) between day 42 and day 0 were examined by ELISA. Data for each donor represent the means of duplicate assays*.

### Efficacy of nivolumab at 3 months after first administration

Among patients with cutaneous melanoma, complete response (CR) was seen in 1 patient (1.7%; 95% confidence interval [CI], 0–3.4%), partial response (PR) in 12 patients (20.3%; 95%CI, 0–40.6%), stable disease (SD) in 16 patients (27.1%; 95%CI, 0–54.2%), and progressive disease (PD) in 29 patients (49.2%; 95%CI, 0–98.4%). The objective response rate (ORR) at 3 months after first administration was thus 22.0% (95%CI, 0–44.0%). Tumor responses of individual patients are listed in Table 1. Among patients with mucosal melanoma, PR was seen in 4 patients (25.0%; 95%CI, 0–50.0%), SD in 5 patients (31.3%; 95%CI, 0–62.6%), and PD in 7 patients (43.8%; 95%CI, 0–87.6%). The ORR at 3 months after first administration was thus 25.0% (95%CI, 0–50.0%). Tumor responses of individual patients are listed in Table 2.

### Serum levels of sCD163

**Figure 1:**
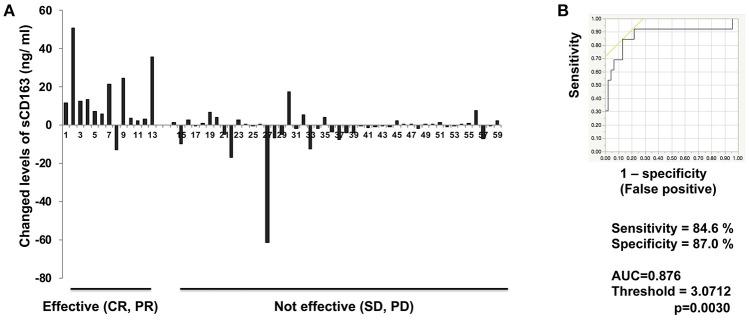
Serum levels of sCD163 and ROC curve in cutaneous melanoma. Serum level changes of sCD163 in each patient (*n* = 59) **(A)** on day 42. The ROC curve was applied to calculate cut-offs for sCD163 serum levels and AUC **(B)**. Cut-offs were determined to distinguish responders from nonresponders using Youden's index.

**Figure 2:**
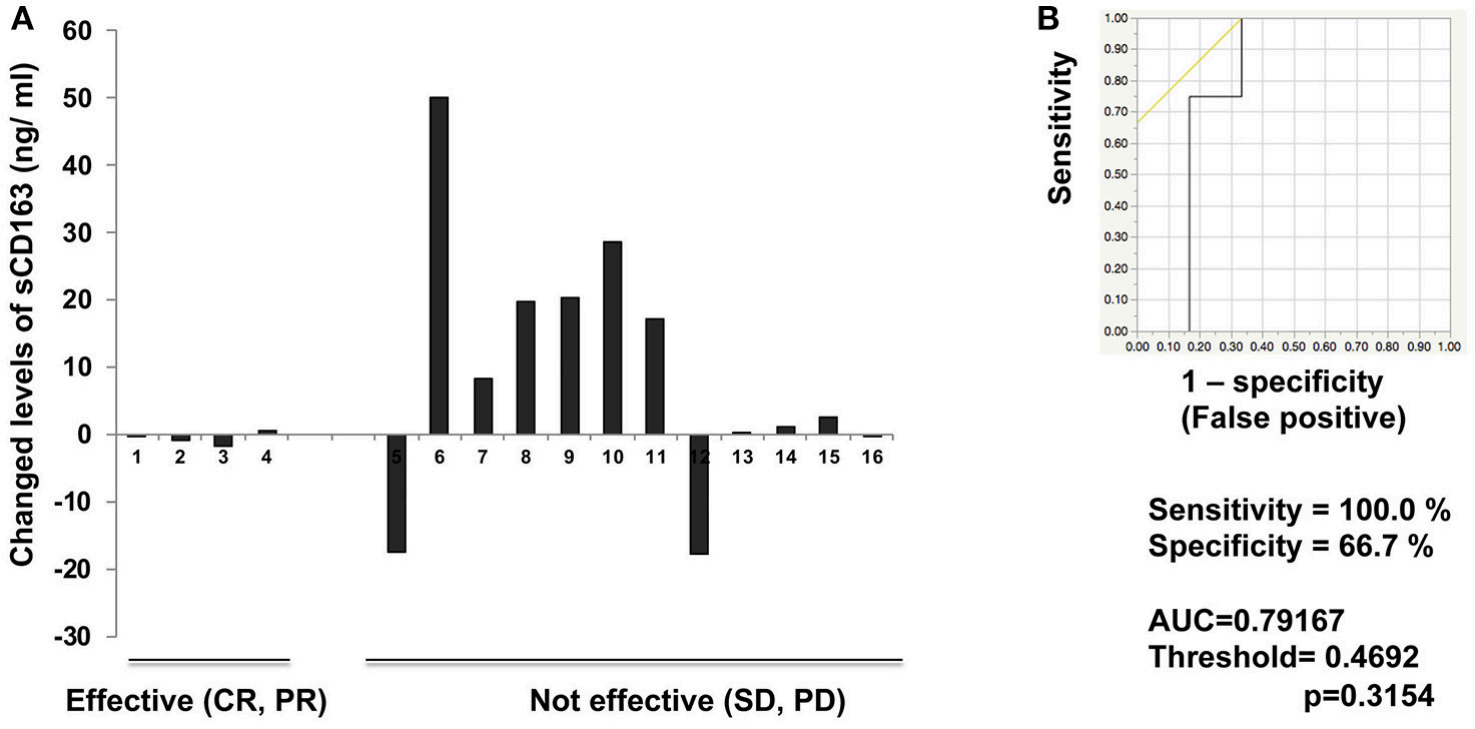
Serum levels of sCD163 and ROC curve in non-cutaneous melanoma. Serum level changes of sCD163 in each patient (*n* = 16) **(A)** on day 42. The ROC curve was applied to calculate cut-offs for sCD163 serum levels and AUC **(B)**. Cut-offs were determined to distinguish responders from nonresponders using Youden's index.

To determine whether serum levels of sCD163 may predict early response in melanoma patients treated with nivolumab, we evaluated levels in 75 patients with advanced melanoma treated using nivolumab (Supplemental Figure 1). Compared to baseline (day 0), serum levels of sCD163 at day 42 were significantly increased in the group showing objective response (*p* < 0.0001; Figure 1A) among patients with advanced cutaneous melanoma, whereas no significant difference in serum sCD163 levels was seen among patients with advanced mucosal melanoma (Figure 2A). Increases in serum sCD163 and efficacy at 3 months after the first administration of nivolumab in each patient are described in Tables 1, 2. The thresholds for sCD163 change between day 42 and day 0 to distinguish responders from non-responders were respectively 3.07 ± 0.07 ng/mL and 0.47 ± 0.05 ng/mL in cutaneous and non-cutaneous melanoma. In the cutaneous melanoma cohort, the mean change ratio in sCD163 serum level was 144.6% for those patients showing objective response, compared to 101.0% for non-responders (Table 1). In the mucosal melanoma cohort, the mean change ratio in sCD163 serum level was 84.1% for those patients showing objective response, and 122.3% for non-responders (Table 2). The threshold for increased serum sCD163 in cutaneous melanoma was 3.07 ± 0.07 ng/mL, whereas that in non-cutaneous melanoma was 0.47 ± 0.05 ng/mL. The sensitivity and specificity of serum sCD163 in cutaneous melanoma were 84.6 and 87.0%, respectively (*p* = 0.0030; Figure 1B), whereas the sensitivity and specificity of serum sCD163 in non-cutaneous melanoma were 100 and 66.7%, respectively (*p* = 0.3154; Figure 2B).

## Discussion

Because nivolumab shows higher efficacy than other anti-melanoma drugs (e.g., ipilimumab and dacarbazine) (1, 17), and induces a longer duration of anti-tumor response than BRAF/MEK inhibitors (e.g., vemurafenib, dabrafenib, trametinib) (18, 19), oncologists have been particularly interested in combining nivolumab with agents that enhance anti-tumor immune responses in patients with metastatic melanoma (1–3, 20). The efficacy of nivolumab appears significantly increased when combined with ipilimumab (57.7%), but the rate of severe treatment-related AEs (Grade 3 or 4) is unfortunately also significantly increased with this particular combination (59.0%) (1, 2). To avoid severe AEs caused by ipilimumab, predictive biomarkers are needed to evaluate the efficacy of nivolumab monotherapy at 2–3 months after first administration, to prepare for any planned switch from nivolumab to ipilimumab.

CD163 is a member of the scavenger receptor cysteine-rich family, and is exclusively expressed on cells of the monocyte/macrophage lineage (15). As previously reported, in metastatic melanoma, a substantial number of CD163^+^ TAMs are present in metastatic melanoma and are activated by stromal factors, leading to an increase in serum levels of sCD163 as a result of proteolytic shedding (21–23). In addition, Jensen et al. (21) previously reported sCD163 and CD163^+^ TAMs as a prognostic marker for early-stage cutaneous melanoma. Notably, as Gordon et al. (14) reported, PD-1 expression is a key factor in maintaining TAMs as M2-polarized, and blockade of PD-1/PD-L1 leads to conversion of TAMs into M1-polarized activated macrophages. Because TAMs in melanoma comprise a heterogeneous, mainly immunosuppressive population (9, 23), and because repolarization of TAMs into the activated subtype by immunomodulatory drugs has been reported to significantly suppress melanoma growth in a spontaneous mouse melanoma model (8), we hypothesized that one of the targets for nivolumab in melanoma is activated CD163^+^ TAMs.

To prove our hypothesis, we analyzed serum levels of sCD163 in 59 cases of advanced cutaneous melanoma and 16 cases of advanced mucosal melanoma treated with nivolumab. Serum levels of sCD163 were significantly increased 6 weeks after initial administration of nivolumab in the response group compared to the non-response group in cutaneous melanoma. In contrast, no significant difference between nivolumab responders and non-responders was seen for non-cutaneous melanoma. Interestingly, in non-cutaneous melanoma, serum levels of sCD163 in non-responders tended to be even higher than those in responders. This discrepancy might be explained by differences in cancer stroma that could stimulate TAMs in the tumor microenvironment of each organ. Such studies should be performed in the future.

According to the present results, sCD163 may represent a predictive biomarker for evaluating the efficacy of nivolumab at 3 months after first administration for advanced cutaneous melanoma. Because sequential administration of ipilimumab followed by nivolumab is only effective for advanced melanoma before the melanoma develops tolerance for nivolumab (7), and administration of ipilimumab leads to the development of irAEs more frequently than nivolumab (2, 3), predicting the efficacy of nivolumab before first tumor estimation is crucial to determining whether the patient will successfully adapt to the planned switch from nivolumab to ipilimumab therapy. The present study suggested that sCD163 may be a useful biomarker for the selection of those cutaneous melanoma patients most likely to benefit from anti-melanoma immunotherapy using nivolumab and ipilimumab. Because this was a pilot study, future independent studies with larger patient cohorts are needed to confirm our findings.

## Author contributions

TFuj designed the research study. TFuj, YS, KT, and YK performed and analyzed the ELISA data. TFuj, YK, AO, YF, KY, SM, TFun, HH, YY, HU, YN, RT, MA, KI, HO, NW, HI, TH, and AH treated the patients and acquired the clinical data and samples. TFuj wrote the manuscript. TFuj and SA supervised the study.

### Conflict of interest statement

The authors declare that the research was conducted in the absence of any commercial or financial relationships that could be construed as a potential conflict of interest.

## Supplementary material

Supplemental Figure 1 Serum levels of sCD163 at day 0 in patients with cutaneous and non-cutaneous melanoma. Mean serum levels of sCD163 in responders (*n* = 13) and non-responders (*n* = 46) at day 0 **(A)**. Mean serum levels of sCD163 in responders (*n* = 4) and non-responders (*n* = 12) at day 0 **(B)** n.s, not significant.
