# Supplementary figures and images for: Serum Level of Soluble CD163 May Be a Predictive Marker of the Effectiveness of Nivolumab in Patients With Advanced Cutaneous Melanoma
Source: Front Oncol. 2018 Nov 19;8:530. doi: 10.3389/fonc.2018.00530 (PMC6252386; doi:10.3389/fonc.2018.00530)

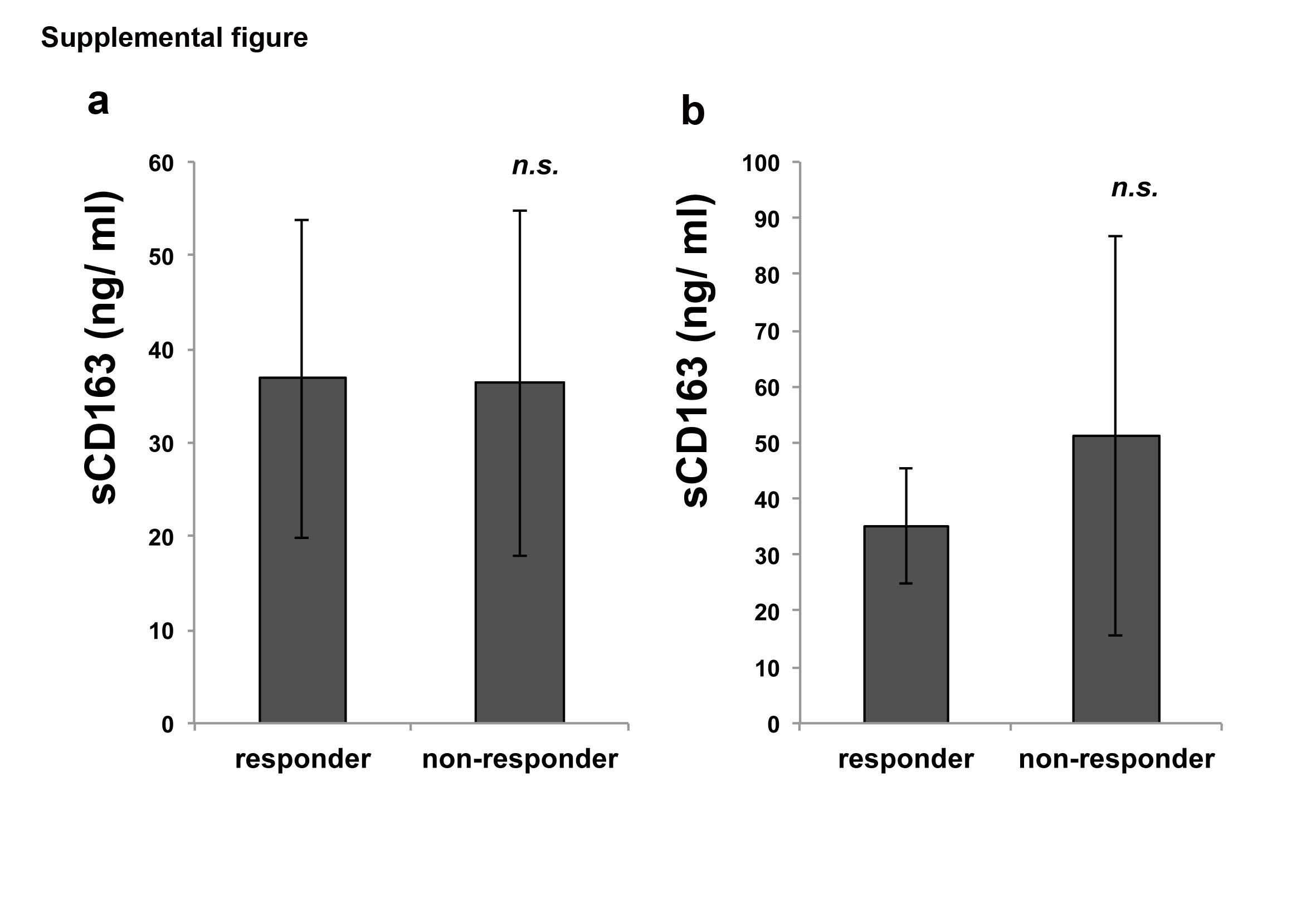

Supplement: Supplemental Figure 1 — Serum levels of sCD163 at day 0 in patients with cutaneous and non-cutaneous melanoma. Mean serum levels of sCD163 in responders (n = 13) and non-responders (n = 46) at day 0 (A). Mean serum levels of sCD163 in responders (n = 4) and non-responders (n = 12) at day 0 (B) n.s, not significant. [file Image_1.JPEG]
